# ABO blood groups and expression of blood group antigens of epithelial ovarian cancer in Chinese women

**DOI:** 10.1002/cam4.5476

**Published:** 2022-11-22

**Authors:** Chao Wang, Jingjing Zhou, Lili Wang, Tongyu Xing, Hongji Dai, Yao Zhou, Lisha Qi, Yanrui Zhao, Caiyun Huang, Ding Li, Haixin Li, Mulin Jun Li, Ben Liu, Hong Zheng, Kexin Chen, Lian Li

**Affiliations:** ^1^ Department of Epidemiology and Biostatistics Tianjin Medical University Cancer Institute and Hospital, National Clinical Research Center for Cancer, Key Laboratory of Cancer Prevention and Therapy of Tianjin, Tianjin's Clinical Research Center for Cancer, Key Laboratory of Molecular Cancer Epidemiology of Tianjin Tianjin China; ^2^ Department of Pharmacology, the Province and Ministry Co‐sponsored Collaborative Innovation Center for Medical Epigenetics, School of Basic Medical Sciences Tianjin Medical University Tianjin China; ^3^ Department of Pathology Tianjin Medical University Cancer Institute and Hospital, National Clinical Research Center for Cancer, Key Laboratory of Cancer Prevention and Therapy of Tianjin, Tianjin's Clinical Research Center for Cancer Tianjin China; ^4^ Department of Clinical Laboratory Tianjin Medical University Cancer Institute and Hospital, National Clinical Research Center for Cancer, Key Laboratory of Cancer Prevention and Therapy, Tianjin's Clinical Research Center for Cancer Tianjin P. R. China; ^5^ Cancer Biobank Tianjin Medical University Cancer Institute and Hospital, National Clinical Research Center for Cancer, Key Laboratory of Cancer Prevention and Therapy of Tianjin, Tianjin's Clinical Research Center for Cancer Tianjin China

**Keywords:** ABH antigens, ABO blood group, epithelial ovarian cancer, risk

## Abstract

**Background:**

ABO blood groups has been associated with risk of several cancers; however, the results for an association with ovarian cancer are inconsistent and little is known about the expression of histo‐blood group (ABH) antigens and *ABO* gene in ovarian tumor tissues.

**Methods:**

To assess the impact of genotype‐derived ABO blood types on the risk of EOC, we conducted a case–control study in 1,870 EOC and 4,829 controls. Expression of A and B antigen in 70 pairs of ovarian tumor tissues and adjacent normal tissues were detected by immunohistochemistry. Gene expression and DNA methylation profiling was conducted in ovarian tumor tissues.

**Results:**

We identified that blood group A was associated with increased risk for EOC compared to blood group O (*OR* = 1.18, 95% CI = 1.03–1.36, *p* = 0.019). Increased frequency of aberrant expression of histo‐blood group antigens was observed in patients with blood group A (76.5%) compared to patients with blood group O (21.1%) and B (5.0%) by immunohistochemistry (*p* < 0.001). *ABO* gene expression was down‐regulated in ovarian tumor tissues compared with paired adjacent normal tissues (*p* = 0.027). In addition, *ABO* gene expression was positively correlated with *NFYB* (*r* = 0.38, *p* < 0.001) and inversely correlated with DNA methylation level of four CpG sites on ABO gene (cg11879188, *r* = − 0.3, *p* = 0.002; cg22535403, *r* = − 0.30, *p* = 0.002; cg13506600, *r* = − 0.22, *p* = 0.025; cg07241568, *r* = − 0.21, *p* = 0.049) in ovarian tumor tissues.

**Conclusion:**

We identified blood group A was associated with increased EOC risk in Chinese women and provided the clues of the possible molecular mechanisms of blood group A related to ovarian cancer risk.

## Abstract 2

We identified blood group A was associated with increased EOC risk in Chinese women. We provided the clues of the possible molecular mechanisms of blood group A related to ovarian cancer risk.

## 1 INTRODUCTION

The ABO blood group is the most important human blood group system which was classified into four groups, A, B, AB, and O, according to the presence of carbohydrate antigen A and antigen B on red blood cells. The antigen A and antigen B was synthesized from the precursor substrate H substance by an addition of specific sugar residues, respectively.^1^ They were catalyzed by N‐acetylgalactosamine transferase and galactosamine transferase, which were encoded by *ABO* gene located at 9q34.2.^2^ It has been reported that these antigens are expressed not only on the surface of red blood cells, but also on cells of other tissues such as epithelium.^3^ And aberrant expression of blood group antigen was observed in carcinomas of the stomach, proximal colon, pancreas, and lung, etc.^4^ ABO blood group system has been reported to be associated with several diseases, including COVID‐19, pancreatic cancer, gastric cancer, and ovarian cancer etc.^5^, ^6^, ^7^

Ovarian cancer is the seventh most common cancer and the second most common cause of gynecologic cancer death worldwide.^8^ In China, the incidence rate of ovarian cancer has increased sharply recently.^9^ The evidence for association between ABO blood group and ovarian cancer is inconsistent. A number of case–control studies and meta‐analysis suggested that blood group A was associated with ovarian cancer risk compared to the O blood group.^10^, ^11^, ^12^, ^13^, ^14^ However, the increased risk of ovarian cancer was reported to be associated with the B allele in a recent cohort study.^15^ The underlying mechanisms for the association of ABO blood groups with cancer risk were unclear and aberrant expression of blood group antigen may be one of the mechanisms that were related to carcinogenesis of ovarian cancer. Research on the expression of ABH antigen in ovarian cancer tissues has remained limited to date.^16^, ^17^ Therefore, the studies to reveal the association between ABO blood group and ovarian cancer risk and the underling molecular mechanisms were needed.

Here, we conducted a case–control study to evaluate the associations between ABO blood groups and the risk of epithelial ovarian cancer (EOC). The expression of histo‐blood group antigens and *ABO* gene expression in ovarian tumor tissues versus adjacent normal tissues were evaluated. In addition, we examined the relationship between *ABO* gene expression and transcription factors/DNA methylation related to *ABO* gene in ovarian tumor tissues.

## 2 MATERIAL AND METHODS

### 2.1 Study populations and assessment of ABO blood groups

ABO blood group was determined using two single nucleotide polymorphisms (SNPs) on *ABO* gene. The genotype data were obtained from our previous genome‐wide association study. The study design, populations, and genotyping method were described elsewhere.^18^, ^19^, ^20^ Briefly, a total of 1,870 EOC cases and 4,829 cancer‐free controls were included. A total of 1,069 EOC cases of northern Chinese population were recruited from Tianjin, Shandong, and Hebei province and 801 eastern Chinese population was recruited from Shanghai and Jiangsu province. Controls (2,761 cancer free women for northern and 2,068 cancer free women for eastern) were randomly selected and were frequently matched to cases by age (5 year group) and area. The characteristics of the participants are summarized in Table 1. ABO blood groups were inferred by estimating haplotypes of two SNPs (rs8176746 and rs687289) in *ABO* gene with >99% posterior probability. The rs8176746 polymorphism is a marker of the B allele, while rs687289 is perfectly correlated with rs8176719 (*r* ^2^ = 1), a marker of the O allele. For determining the haplotype phase, we refer to the analysis method previously reported by Mao et al.^21^ The information of age and area was obtained for all participants. Demographic information and clinical characteristics of participants in Tianjin were obtained by baseline questionnaire (BMI, first‐degree family cancer history, age of menarche, no. of children, menopause, tubal ligation). Informed consent was obtained from each subject, and this study was approved by the Institutional Review Board of Tianjin Medical University Cancer Institute and Hospital study.

**TABLE 1:** Baseline information and clinical features of study subjects included for logistic regression analysis

	Northern			Eastern			Total		
	Case	Control	*p*	Case	Control	*p*	Case	Control	*p*
*N*	1069	2761	—	801	2068	—	1870	4829	—
Age^a^	53.76 ± 11.01	53.48 ± 7.72	0.456	54.10 ± 10.18	54.41 ± 10.25	0.477	53.9 ± 10.66	53.87 ± 8.91	0.890
Blood group^b^									
O	296 (27.7)	812 (29.4)		214 (26.7)	605 (29.3)		510 (27.3)	1417 (29.3)	
A	295 (27.6)	707 (25.6)		277 (34.6)	635 (30.7)		572 (30.6)	1342 (27.8)	
B	344 (32.2)	923 (33.4)		226 (28.2)	617 (29.8)		570 (30.5)	1540 (31.9)	
AB	134 (12.5)	319 (11.6)		84 (10.5)	211 (10.2)		218 (11.7)	530 (11.0)	
Histology^b^			—			—			—
Serous	506 (47.3)	—		500 (62.4)	—		1006 (53.8)	—	
Mucinous	81 (7.6)	—		38 (4.7)	—		119 (6.4)	—	
Endometrioid	258 (24.1)	—		49 (6.1)	—		307 (16.4)	—	
Clear Cell	16 (1.5)	—		33 (4.1)	—		49 (2.6)	—	
Others	208 (19.5)	—		181 (22.6)	—		389 (20.8)	—	

^a^ Mean ± SD.

^b^ *N* (%).

### 2.2 Tissue microarray construction

The tissue microarray was constructed from patients diagnosed with primary EOC at the Tianjin Medical University Cancer Hospital between January 2012 and November 2013. At least two cores of tumors and one core of matched adjacent non‐tumor tissues were collected from each patient following surgery, then paraffin‐embedded and cut into 4‐mm sections for immunohistochemical staining. Cases without adjacent non‐tumor tissues or ABO blood group record were excluded. A total of 70 samples were included in this study.

### 2.3 Immunohistochemical staining and evaluation

The blood group A antigen monoclonal antibody (MA1‐19693; dilution 1:200, Thermo Scientific, Pierce Protein Research) and the blood group B antigen monoclonal antibody (MA1‐19691; dilution 1:100, Pierce Protein Research) have been used. Immunohistochemical staining was carried out by using a two‐step protocol. Briefly, tissue microarray slides were dried 60 min at 60°C and then were deparaffinized and rehydrated. Antigen retrieval was performed using citrate buffer (pH 6.0) in a pressure cooker. Slides were incubated overnight at 4°C with primary antibody and secondary antibody (K5007; DakoCytomation) for 30 min. The slides then were stained with 3,3‐diaminobenzidine tetrahydrochloride (ZLI‐9017; ZSGB‐BIO) and counterstained with Mayer hematoxylin (Klinipath B.V.).

Brown cytoplasmic and / or membrane staining was counted as positive. Immunohistochemistry results were evaluated with a semi‐quantitative method and given a score between 0 and 7. The scores were calculated as the intensity score (0, negative; 1, weak; 2, moderate; 3, high) plus the percentage of cells (0, no staining; 1, 1–25% positive cells; 2, 26–50% positive cells; 3, 51–75% positive cells; 4, 76–100% positive cells). Scores were considered negative (0), weakly positive,^1^, ^2^, ^3^ moderately positive,^4^, ^5^ and strongly positive.^6^, ^7^ Immunohistochemical staining was independently scored by two people who were blinded to the clinical data at the time of assessment.

### 2.4 Gene expression analysis

Expression profiling data of ovarian tumor tissues and paired normal tissues was obtained from our previous study.^22^ ABO blood groups of 14 high‐grade serous ovarian patients were obtained from the electronic medical records. ABH antigen synthesis‐related genes in the KEGG “Glycosphingolipid biosynthesis‐lacto and neolacto series” pathway were included in subsequent analyses. Transcription factors related to *ABO* gene were also included for subsequent analysis.^23^

The expression of *ABO* gene and related transcription factors in ovarian tumor tissues was measured by RNA‐sequencing as previously reported.^24^ In brief, 116 ovarian tumor tissues were acquired from the tumor tissue bank of Tianjin Medical University Cancer Institute and Hospital (TMUCIH). Total RNA was extracted using standard TRIZOL method. Sequencing libraries were generated using NEBNext® UltraTM RNA Library Prep Kit for Illumina® (NEB, USA) following the manufacturer's recommendations. Sequencing was conducted with the Illumina NovaSeq 6000 platform and 150‐bp paired‐end reads were generated. Read alignment was performed using STAR 2.5.3a and calculated expected transcript per million (TMP) using RSEM 1.3.0.

### 2.5 DNA methylation analysis

Matched gene expression and DNA methylation data were available for 112 cases.^24^ DNA methylation profiling was conducted using Illumina Infinium Human MethylationEPIC (850 K) BeadChip as previously reported.^24^ Data were imported and normalized using the ChAMP R package. The raw intensity files were loaded with all probes passing the detection *p* ≤ 0.01 and filtered to exclude the probes with a beadcount <3 in at least 5% of samples or targeting CpG with a SNP. The beta values were normalized using BMIQ and afterward the batch effects were identified and removed using combat. The CpG sites were annotated to genes using 850K annotation resources. CpG located in 2 kb upstream and downstream of *ABO* gene were included in the subsequent analyses.

### 2.6 Statistical analyses

The differences in the distributions of demographic characteristics between groups were assessed using the Student's *t*‐test for continuous variables and the Pearson's *χ* ^*2*^ test for categorical variables. To reduce the bias from spatial disparities between different geographic units, the northern population and eastern population was analyzed separately. Main analyses were conducted for both blood group (A, AB, B vs. O) and diplotype (A/A, A/O, A/B, B/B, B/O vs. O/O). Odds ratio (*OR*) and 95% confidence intervals (*CI*) were determined using unconditional logistic regression with adjustment for age in northern populations and eastern populations, respectively. Meta‐analysis was used to combine the results of the northern and eastern populations. A fixed‐effects model was used when the Cochran's Q statistics showed no heterogeneity (*p* > 0.05); otherwise, a random‐effects model was applied. Multivariate logistic regression analysis was performed in Tianjin participants to analyze whether ABO blood group is an independent risk factor of ovarian cancer, and all variables with *p* values less than 0.05 in the univariate analysis were included in the multivariate models. The paired *t*‐test was used to evaluate the differential expressed of ovarian malignant tissues and matched normal tissues of ABO and synthesis‐related genes. Outliers were defined as having values outside of quartile 1–1.5 × interquartile range (IQR) and quartile 3 + 1.5 × IQR. After removing outliers of gene expression and beta value of CpG sites, the Spearman correlation analysis was used to test the association between methylation / ABO related transcription factors and expression of *ABO* gene. All statistical analyses were performed using SPSS 23.0, R software version 4.0.5 and STATA 15.0. Two‐sided *p* values less than 0.05 were considered statistically significant.

## 3 RESULTS

### 3.1 Association between ABO blood group and EOC risk

**FIGURE 1:**
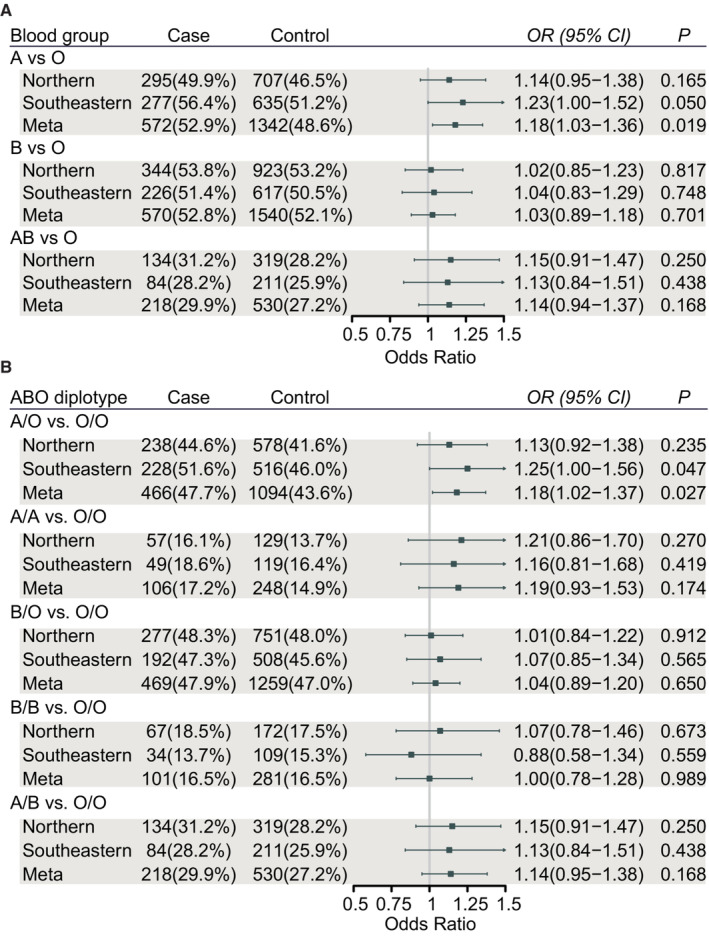
ABO blood group and risk of EOC. Association between ABO blood group (A) and ABO diplotype (B), and EOC risk.

The study included 1,870 EOC cases and 4,829 age‐matched healthy controls (1,069 cases and 2,761 controls from northern area of China, 801 cases and 2,068 controls from eastern China) (Table 1). The genotype distributions of ABO blood groups in ovarian cancer cases and controls are shown in Table S1. No apparent deviations from Hardy–Weinberg equilibrium were observed for rs8176746 (*p* = 0.815 in northern Chinese, *p* = 0.965 in eastern Chinese) and rs687289 (*p* = 0.453 in northern Chinese, *p* = 0.995 in eastern Chinese) among controls. The distribution of blood group O, A, B, and AB was 29.4%, 25.6%, 33.4%, and 11.6% in northern Chinese, and 29.3%, 30.70%, 29.8%, and 10.2% in eastern Chinese, respectively (Table 1). The concordance between agglutination blood group and genotyped blood group in our population was 99.55% (Table S2). We estimated the risk of EOC according to genotype‐derived ABO blood group among all study participants. As shown in Figure 1A, the fixed‐effects meta‐analysis showed participants with A blood group had increased EOC risk compared with those with blood group O (*OR*_meta_ = 1.18, 95% CI = 1.03–1.36, *P*_meta_ = 0.019, *P* for Q = 0.601). When examining the associations with diplotype, compared with subjects with genotype O/O, those with genotype A/O had *OR* of 1.18 (1.02–1.37), *P*_meta_ = 0.027. There was no statistical difference in risk of EOC between A/A and O/O (Figure 1B).

Furthermore, multivariate logistic regression analysis was performed on 452 cases and 2,761 controls in Tianjin whose baseline information was available (Table S3). As expected, higher proportions of nullipara and lower proportions of individuals underwent tubal ligation were noted for cases compared with controls. After adjusting for number of children and tubal ligation, A blood group was still a risk factor for EOC when O blood group was used as the reference (adjusted *OR*  =  1.48, 95% CI: 1.11–1.96, *p* = 0.007) (Figure S1).

### 3.2 Expression differences of histo‐blood group antigens in ovarian tumor tissues and adjacent normal tissues

**FIGURE 2:**
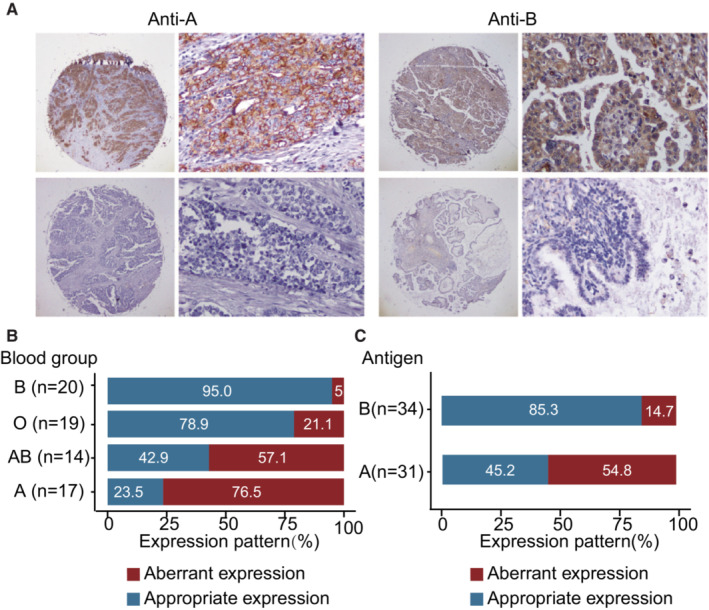
Expression differences of histo‐blood group antigens in ovarian tumor tissues and adjacent normal tissues. (A) Immunohistochemistry showing A antigen maintained expressed (upper left) and loss expression of A antigen (lower left) in ovarian tumor tissue of blood group A. (B) antigen maintained expressed (upper right) and loss expression of B antigen (lower right) in ovarian tumor tissues of blood group B. (B) Proportion of patients maintained or aberrant expression of appropriate antigen according to blood group type. (C) Proportion of patients maintained or loss expression of appropriate antigen according to antigen (Antigen A: patients with blood group A or AB; antigen B: patients with blood group B or AB).

Immunohistochemistry (IHC) was applied to measure the expression of A and B antigens in pairs of normal epithelia of the ovarian tissue and tumor tissues from the 70 patients of blood groups O (*n* = 19), A (*n* = 24), B (*n* = 20), and AB (*n* = 14) (Tables S4 and S5). The frequency of age of menarche, menopause status, histological subtypes, and FIGO tumor stage etc. were balanced between the blood groups, apart from age (*p* = 0.018) (Table S4). A and B antigens were found to be predominantly localized in cytoplasm and cell membrane (Figure 2A). Loss expression of A \ B antigens and incompatible B antigen expression was observed in tumor tissues and these changes were defined as aberrant expression (Figure 2A). The aberrant expression rate in people of the blood group O, A, B, and AB were 21.1%, 76.5%, 5.0%, and 57.1%, respectively. Increased frequency of aberrant expression was observed in patients with blood group A (76.5%) compared to blood group O and blood group B (Figure 2B). The loss rate of A antigen (54.8%) was higher than the loss rate of B antigen (14.7%) (Figure 2C). The expression of A and B antigen was reduced but not completely loss in some of the ovarian tumor tissues. When this change was also defined as aberrant expression, the aberrant expression rate is also higher in patients with blood group A (82.4%) and the loss or reduced rate of A antigen (67.7%) is also higher than B antigen (32.4%) (Tables S6 and S7).

### 3.3 Expression differences in genes related to ABH antigen expression

**FIGURE 3:**
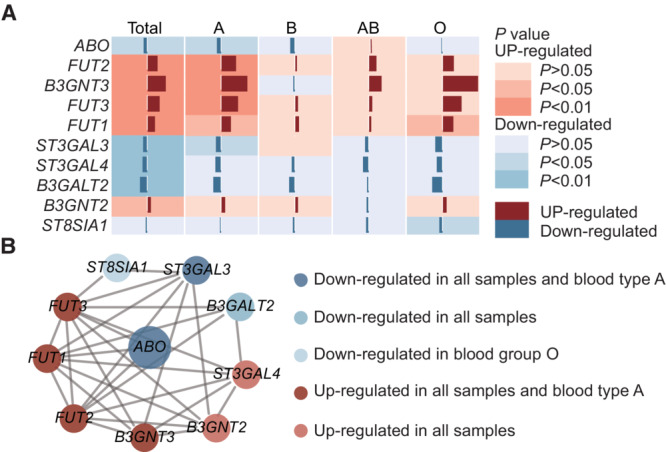
Expression differences in genes related to ABH antigen expression. (A) Differential expression of *ABO* gene and ABH antigen synthesis‐related genes between ovarian cancer tissues and adjacent normal tissues. (B) The protein–protein interactions of differential expression genes involved in ABH antigen synthesis.

*ABO* gene expression was down‐regulated in ovarian tumor tissues compared with adjacent normal tissues (*p* = 0.013). In particular, only the reduction in seven blood group A samples was statistically significant (*p* = 0.027) (Figure 3A, Table S8 and S9). As ABH antigens are terminal structures of glycan chain, glycosyltransferases which involved in precursor structure may affect the synthesis of ABH antigen. We observed 24 ABH antigen synthesis‐related genes in which five genes (*FUT1*, *FUT2*, *FUT3*, *B3GNT3*, and *B3GNT2*) were up‐regulated and three genes (*ST3GAL3*, *ST3GAL4*, and *B3GALT2*) were down‐regulated in ovarian tumor tissue versus adjacent tissues in all samples. Of these, *B3GNT3*, *FUT1*, *FUT2*, and *FUT3* were up‐regulated and *ST3GAL3* was down‐regulated in blood group A patient (Figure 3B, Table S9).

### 3.4 Correlation between *ABO* gene expression and related transcription factors

**FIGURE 4:**
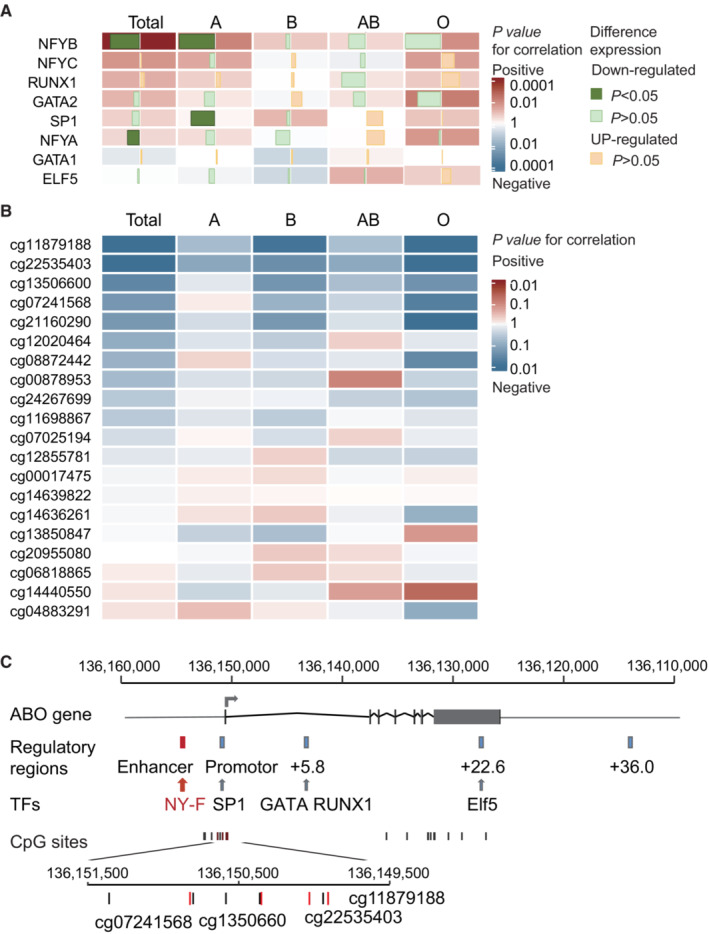
Correlation between *ABO* gene expression and related transcription factors / DNA methylation. (A) Heatmap showing the correlation between related transcription factors and *ABO* gene expression in ovarian tumor tissues. The bars represent the relative expression in ovarian cancer tissues compared with corresponding normal tissue. (B) Heatmap showing the correlation between CpG sites DNA methylation level and *ABO* gene expression. (C) Schematic description of possible factors affecting expression levels of *ABO* gene.

To identify the possible transcription factors involved in regulation of *ABO* gene expression, we evaluated the correlation between *ABO* gene expression and the transcription factors that have been reported to be related to *ABO* gene expression previously (Table S10). We found that *Sp1* (*p* = 0.004) and *NFYB* (*p* = 0.012) were down‐regulated in blood group A and *NFYA* (*p* = 0.046) was significantly down‐regulated in total (*n* = 14) (Figure 4A, Table S9). Of these, *NFYB* was positively correlated with *ABO* gene expression in 116 patients (*r* = 0.38, *p* < 0.001). A statistically significant positive correlation of *NFYB* and *ABO* gene expression was also observed in patients with A blood group (*r* = 0.43, *p* < 0.001) (Figure 4A, Table S11, Figure S2).

### 3.5 Correlation between *ABO* gene expression and DNA methylation on *ABO* gene

A total of 20 CpG methylation sites on *ABO* gene were evaluated, we found that DNA methylation level of four CpG sites were significantly inversely correlated with *ABO* gene expression (cg11879188, *r* = 0.3, *p* = 0.002; cg22535403, *r* = 0.30, *p* = 0.002; cg13506600, *r* = 0.22, *p* = 0.025; cg07241568, *r* = 0.21, *p* = 0.049) (Figure 4B, Table S12, Figure S3). In addition, two CpG sites were found to be inversely correlated with *ABO* gene expression in individuals with O blood group (cg21160290, *r* = −0.53, *p* = 0.006; cg08872442, *r* = −0.44, *p* = 0.029) (Figure S4). In this study, we provided a possible mechanism that *ABO* gene expression may be regulated by transcription factor *NY‐F* and/or four DNA methylation sites (cg11879188, cg22535403, cg13506600, and cg07241568) (Figure 4C).

## 4 DISCUSSION

In this study, we found that blood group A was significantly associated with increased risk of EOC in Chinese women in 1,870 EOC and 4,829 controls. The increased risk of blood group A remained significant after adjustment for potential confounding factors in 452 cases and 2,761 controls. Increased frequency of aberrant expression of A antigen was observed in ovarian tumor tissues by IHC. We also identified that *ABO* gene expression was lower in ovarian tumor tissues than adjacent normal tissues. Additionally, we first noted that *NFYB* was positively correlated with *ABO* gene expression and DNA methylation (four CpG sites) of *ABO* gene was negatively correlated with *ABO* gene expression.

Previous studies have reported the increased EOC risk associated with the A blood group compared to the O blood group with *OR* range of 1.09–1.40.^11^, ^12^, ^13^, ^14^, ^25^ One study with the NHS cohort showed an increased risk of ovarian cancer among women with any B allele (blood groups B and AB) compared to women with the O blood group (*RR* = 1.41; 95% CI: 1.06–1.88).^15^ Since the information of ABO blood group in most of the previous studies was obtained from questionnaire, the misclassification bias might be existed. Genotype‐derived ABO blood group is highly accurate and also allowed us to evaluate the associations of ABO diplotype with EOC risk. At present, only one study reported genotype‐derived ABO diplotype and risk of EOC in European populations by Ovarian Cancer Association Consortium (OCAC).^12^ The results consistent with our work that the association was only observed in the A/O, but not in the A/A diplotype, may be due to the low frequency of the A/A diplotype may not have enough power to reach the significant result.

Although ABO blood group was reported to be associated with several types of cancers, the underline biological mechanism remains unclear. Recent studies reported the association between ABO variants and pro‐inflammatory molecules (e.g., tumor necrosis factor‐alpha, soluble intercellular adhesion molecule, E‐selectin, P‐selectin, and von Willebrand factor) suggesting that ABO blood group may play a role in the immune systemic response.^26^, ^27^, ^28^, ^29^, ^30^ Another plausible hypothesis encompasses the altered pattern of blood group antigen expression in malignancy.^7^, ^25^, ^31^ ABH antigens are oligosaccharide chains by sequentially adding sugars to the precursor via the corresponding glycosyltransferases encoded by *ABO* gene. In concordance with the results of studies by Metoki and Welshinger, we also found loss expression of A and B antigens in ovarian tumor tissue.^16^, ^17^ Hence, the change of blood group antigens expression may affect cell adhesion, membrane signaling, and immune responses.^32^ Increased frequency of aberrant expression patterns in A blood group may partly explain the increased risk of A blood group. ABO glycosylation in normal and malignant urothelium is regulated at the mRNA level.^33^ We also found *ABO* gene expression was down‐regulated in ovarian tumor tissues compared with paired normal tissues of fourteen high‐grade serous ovarian cancer patients, particularly in blood group A patients.

It was reported that several transcription factors, such as *Sp1*, *NF‐Y*, *Elf5*, and *GATA‐1* could regulate *ABO* gene expression.^23^ In our study, we found the expression level of *NFYB* is decreased in ovarian tumor tissues compared with adjacent normal tissues and positive correlation with expression level of *ABO* gene. The protein encoded by *NFYB* is one subunit of a trimeric complex forming a highly conserved nuclear factor‐Y(NF‐Y) which binds to CCAAT motifs in the enhancer regions of *ABO*.^34^ DNA methylation, SNP, and copy number variations also could be a regulatory factor of *ABO* gene.^18^, ^35^, ^36^, ^37^, ^38^ Regarding DNA methylation, we found DNA methylation levels of four CpG sites located near the promoter region (cg11879188, cg22535403, cg13506600, and cg07241568) were significantly inversely correlated with *ABO* gene expression. The significant correlation between *ABO* gene expression and DNA methylation was observed in all samples and O blood group. On the other hand, the ABO genotype that were used in this study, located at chromosome 9q34.2 and there are several genes (e.g., *SURF2*, *SURF4*, *SURF6*, *ATAMTS13*, and *ATAMTSL2*, etc.) that lies within 1 Mb around the genotype. Therefore, it is possible that the ABO SNPs genotyped here are in linkage disequilibrium with variation in another genetic locus.^12^ Further functional studies are warranted to reveal the molecular mechanisms.

To the best of our knowledge, this is the largest study for evaluating the relationship between ABO blood group and EOC risk in Chinese population. Genotype‐derived blood group was used in this study which could reduce misclassification bias from self‐report blood group and allowed us to evaluate the associations of ABO diplotype with EOC risk. Ovarian cancer risk factors are relatively poorly understood, and this study lends support to ABO association with ovarian cancer risk. In this study, we observed the loss of the corresponding antigen in normal epithelial cells of ovarian para‐cancerous tissues by IHC for the first time. In addition, the mechanism for the relationship between ABO type and ovarian cancer remains almost entirely obscure, so this study is a contribution to the literature to explore this. However, although we provided the possible regulatory mechanisms of *ABO* gene expression, validation studies in another distinct large cohort and in vitro/vivo studies were needed.

## 5 CONCLUSIONS

In this study, we identified that blood group A was associated with increased EOC risk in Chinese populations. The expression of A, B histo‐blood group antigens and *ABO* gene were lower in ovarian tumor tissues than that in their adjacent normal tissues and we also found the possible regulatory factors of *ABO* gene expression. Additional functional studies are warranted to explore the possible molecular mechanisms and the role of ABH antigen in ovarian tumor development and progression.

## AUTHOR CONTRIBUTIONS

**Chao Wang:** Conceptualization (equal); formal analysis (equal); software (equal); visualization (equal); writing – original draft (equal). **Jingjing Zhou:** Investigation (equal); methodology (equal). **Lili Wang:** Data curation (equal); investigation (equal). **Tongyu Xing:** Software (equal). **Hongji Dai:** Funding acquisition (equal); methodology (equal). **Yao Zhou:** Methodology (equal); software (equal). **lisha qi:** Funding acquisition (equal). **Yanrui Zhao:** Investigation (equal). **Caiyun Huang:** Investigation (equal). **Ding Li:** Funding acquisition (equal); resources (equal). **Haixin Li:** Funding acquisition (equal); resources (equal). **Mulin Jun Li:** Methodology (equal). **Ben Liu:** Methodology (equal). **Hong Zheng:** Conceptualization (equal); resources (equal). **Kexin Chen:** Conceptualization (equal); funding acquisition (equal); resources (equal). **Lian Li:** Conceptualization (lead); funding acquisition (equal); project administration (lead); resources (equal); supervision (equal); writing – review and editing (equal).

## FUNDING INFORMATION

This work was supported by the National Key R&D Program of China (2021YFC2500400), the National Natural Science Foundation of China (81,973,113, 82,003,544, and 81,802,080), National Human Genetic Resources Sharing Service Platform (2005DKA21300), The National Key Research and Development program of China: The Net construction of human genetic resource Bio‐bank in North China (2016YFC1201703), and special project of the central government to guide local scientific and Technological Development (18ZYPTSY00030).

## CONFLICT OF INTEREST

The authors have no conflict of interest.

## Supporting information


Appendix S1.

Table S11.
Table S12.

## Data Availability

The data used for detecting expression differences in genes related to ABO was obtained from the GSE133859 (GEO, https://www.ncbi.nlm.nih.gov/gds/). Other datasets used and analyzed during the current study are available within the manuscript and its additional files.
