# Supplementary material for: ABO blood groups and expression of blood group antigens of epithelial ovarian cancer in Chinese women
Source: Cancer Med. 2022 Nov 22;12(6):7498–507. doi: 10.1002/cam4.5476 (PMC10067109; doi:10.1002/cam4.5476)
Supplement: Supplementary file 1 — Appendix S1. [file CAM4-12-7498-s001.pdf]

ABO blood groups and expression of blood group antigens of epithelial ovarian cancer in Chinese women

Additional File Information

Fig. S1 Univariate and multivariate analyses for the relationship between ABO blood group and EOC risk

Fig. S2 Linear correlation graphs showing the correlation between the expression level of NFYB and ABO gene

Fig. S3 Linear correlation graphs showing the correlation between DNA methylation levels of cg11879188, cg22535403, cg13506600, cg07241568 and expression of ABO gene

Fig. S4 Linear correlation graphs showing the correlation between DNA methylation levels of cg21160290, cg08872442 and expression of ABO gene

Table S1 Distribution of ABO blood group genotypes in ovarian cancer cases and controls

Table S2 Agglutination tested blood group vs. genotype-derived blood group

Table S3 Baseline characteristics of ovarian cancers and controls included for multivariate logistic regression analysis

Table S4 Baseline and clinical features of 70 EOC patients for IHC staining of antigen A and B

Table S5 Results of IHC staining of antigen A and B in ovarian tumor and adjacent

normal tissues (n = 70)

Table S6 Number of patients reduced or lost the expression of appropriate antigens according to blood group type

Table S7 Number of patients reduced or lost the expression of appropriate antigens according to antigen (antigen A: patients with blood group A or AB; antigen B: patients with blood group B or AB)

Table S8 Baseline and clinical characteristics of 14 patients for gene expression profile

Table S9 The expression of ABO , ABH antigen synthesis-related genes and transcription factors related to ABO gene in 14 HGSOC tumors and paired adjacent normal tissues

Table S10 Baseline and clinical characteristics of 116 patients for RNA-seq and methylation profile

Table S11 Correlation between ABO gene expression and transcription factors related to ABO gene in ovarian tumor tissues

Table S12 Correlation between ABO gene expression and DNA methylation of ABO gene in ovarian tumor tissues

Fig. S1

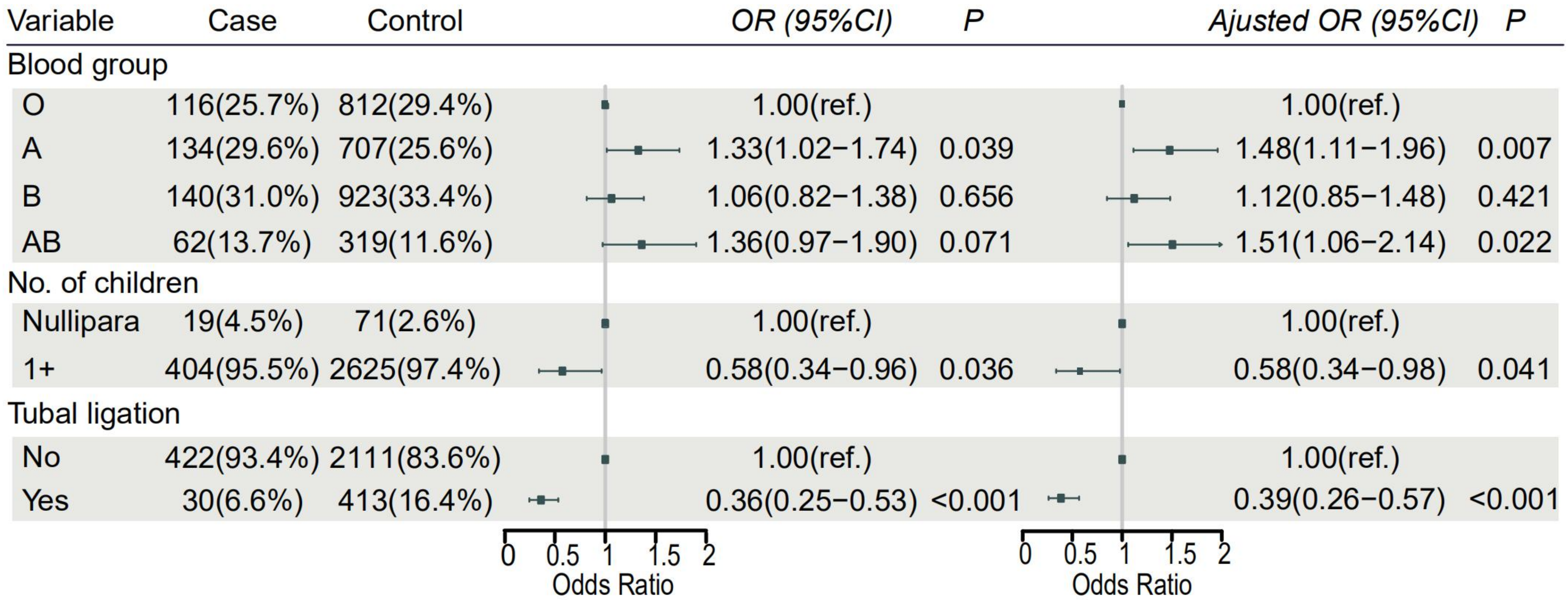

Variable	Case	Control	OR (95%CI)	P	Ajusted OR (95%CI)	P
Blood group						
O	116(25.7%)	812(29.4%)	1.00(ref.)		1.00(ref.)	
A	134(29.6%)	707(25.6%)	1.33(1.02−1.74)	0.039	1.48(1.11−1.96)	0.007
B	140(31.0%)	923(33.4%)	1.06(0.82−1.38)	0.656	1.12(0.85−1.48)	0.421
AB	62(13.7%)	319(11.6%)	1.36(0.97−1.90)	0.071	1.51(1.06−2.14)	0.022
No. of children						
Nullipara	19(4.5%)	71(2.6%)	1.00(ref.)		1.00(ref.)	
1+	404(95.5%)	2625(97.4%)	0.58(0.34−0.96)	0.036	0.58(0.34−0.98)	0.041
Tubal ligation						
No	422(93.4%)	2111(83.6%)	1.00(ref.)		1.00(ref.)	
Yes	30(6.6%)	413(16.4%)	0.36(0.25−0.53)	<0.001	0.39(0.26−0.57)	<0.001

Fig. S1 Univariate and multivariate analyses for the relationship between ABO blood group and EOC risk

Fig. S2

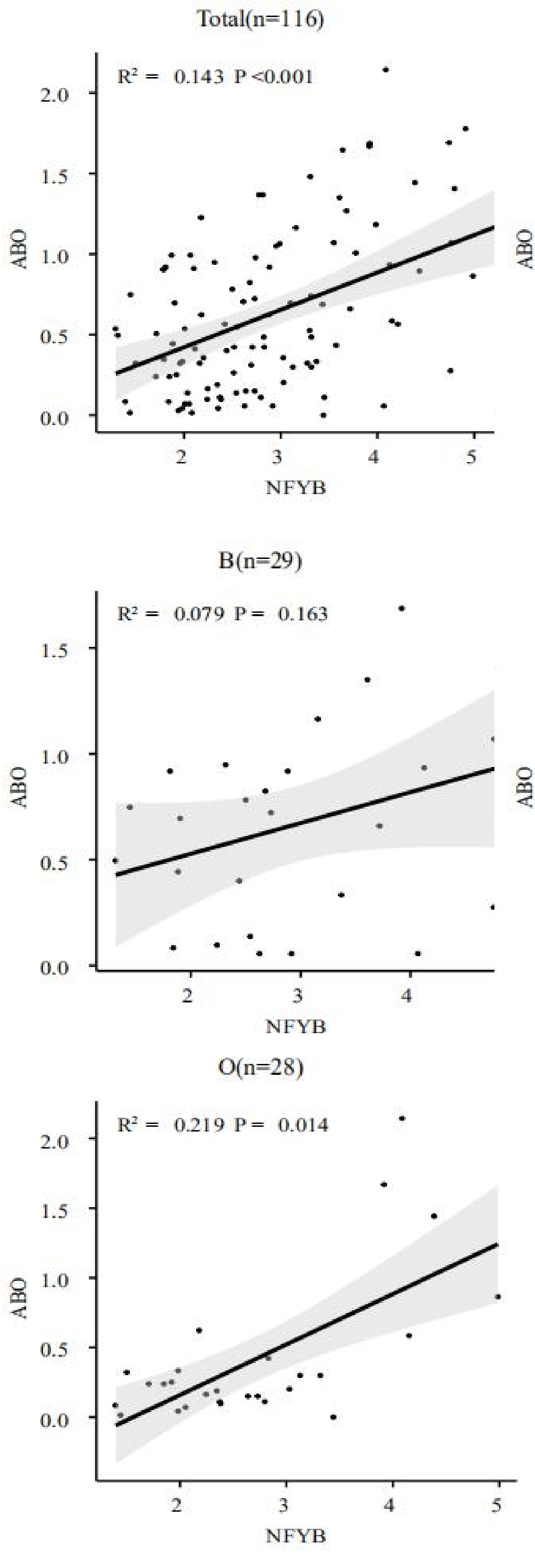

Fig. S2 Linear correlation graphs showing the correlation between the expression level of *NFYB* and *ABO* gene

Fig. S3

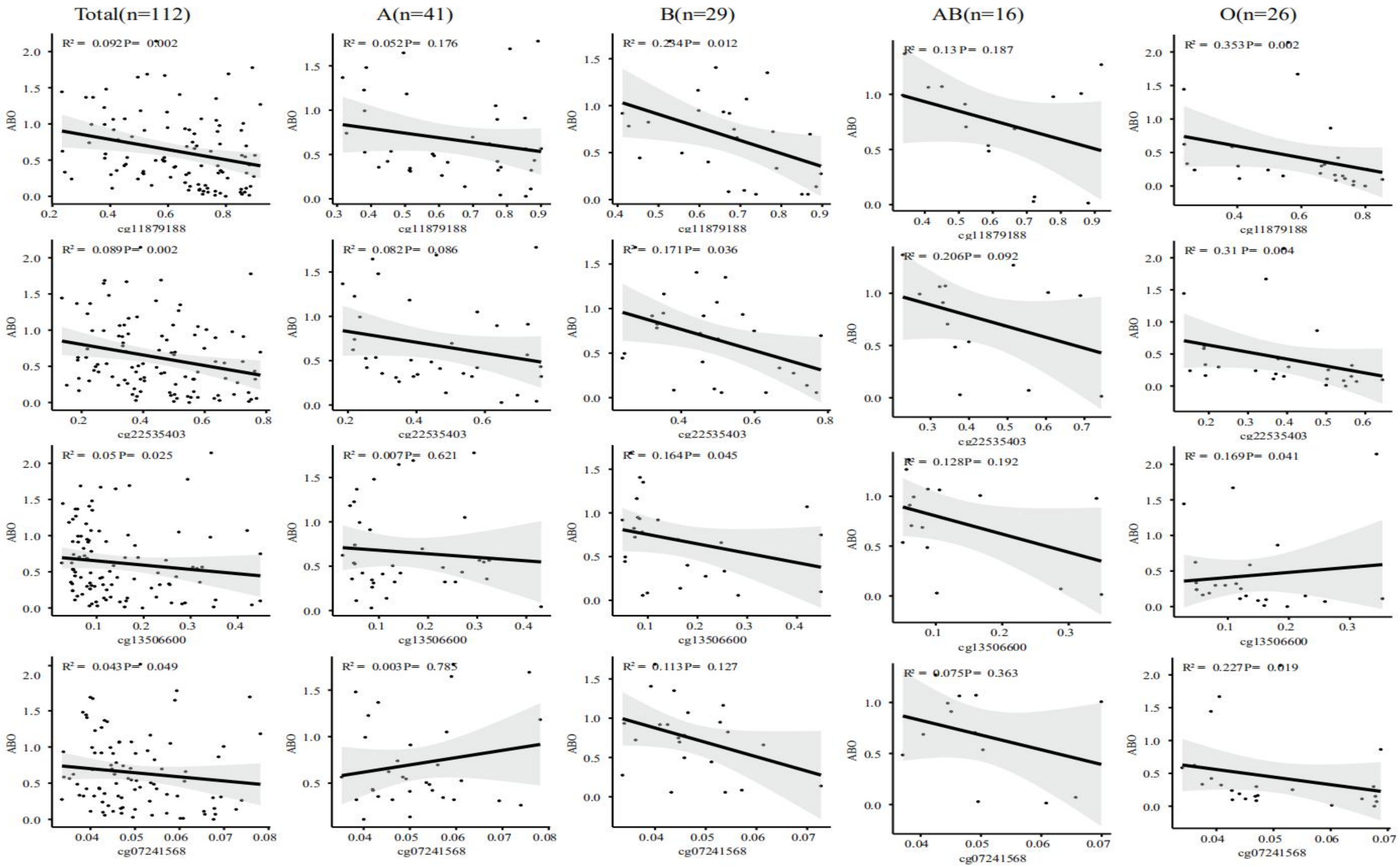

Fig. S3 Linear correlation graphs showing the correlation between DNA methylation levels of cg11879188, cg22535403, cg13506600, cg07241568 and expression of *ABO* gene

Fig. S4

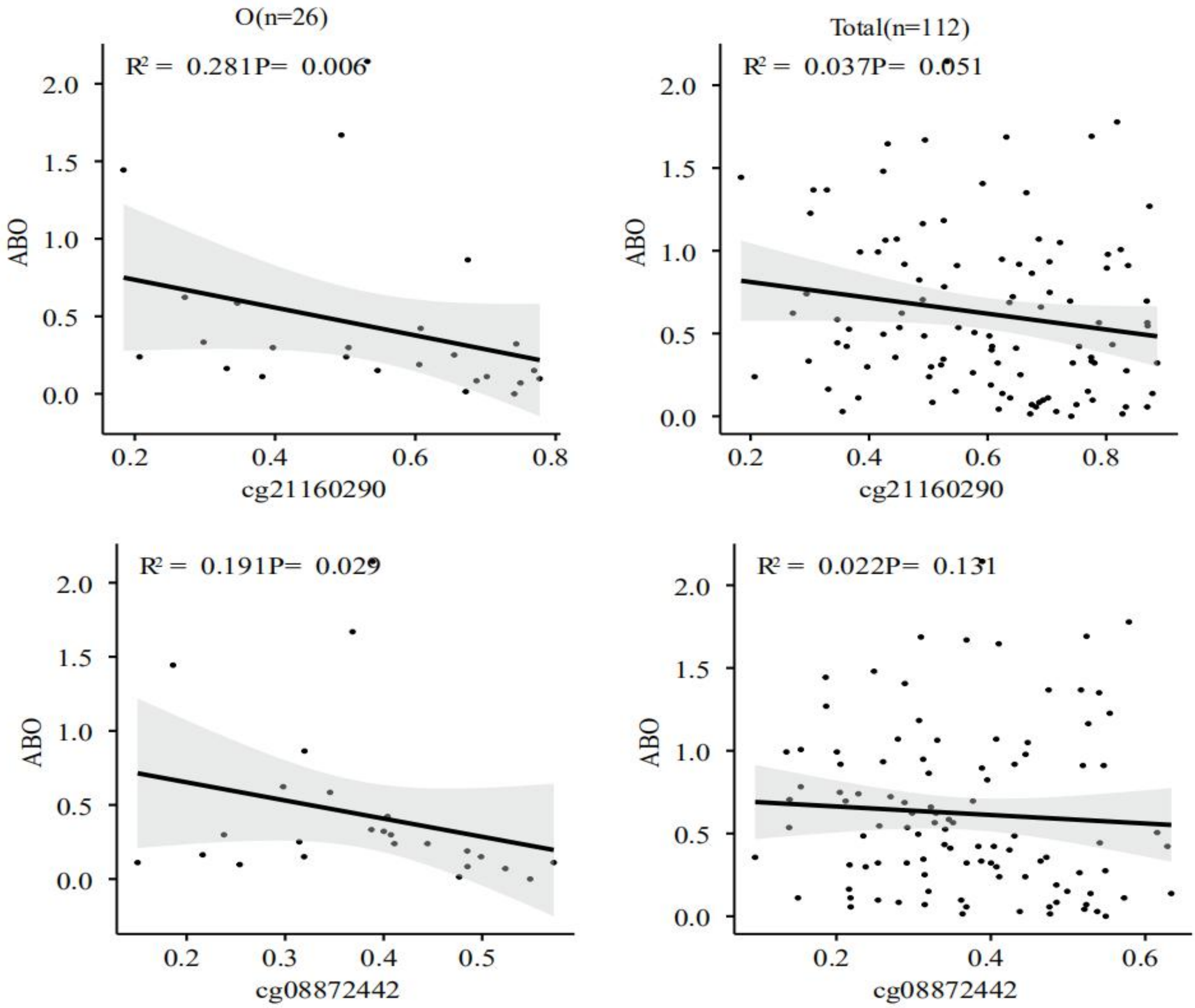

Fig. S4 Linear correlation graphs showing the correlation between DNA methylation levels of cg21160290, cg08872442 and expression of *ABO* gene

Table S1 Distribution of ABO blood group genotypes in ovarian cancer cases and controls

rs687289* rs8176746	Haplotype	Blood group	Northern		Eastern		Total	
			Case	Control	Case	Control	Case	Control
GGGG	OO	O	296(27.7%)	812(29.4%)	214(26.7%)	605(29.3%)	510(27.3%)	1417(29.3%)
AGGG	AO	A	238(22.3%)	578(20.9%)	228(28.5%)	516(25%)	466(24.9%)	1094(22.7%)
AAGG	AA	A	57(5.3%)	129(4.7%)	49(6.1%)	119(5.8%)	106(5.7%)	248(5.1%)
AGTT	BO	B	1(0.1%)	-	2(0.2%)	-	3(0.2%)	-
AATT	BB	B	67(6.3%)	172(6.2%)	34(4.2%)	109(5.3%)	101(5.4%)	281(5.8%)
GGTG	BO	B	1(0.1%)	2(0.1%)	3(0.4%)	-	4(0.2%)	2(0.04%)
AGTG	BO	B	275(25.7%)	749(27.1%)	187(23.3%)	508(24.6%)	462(24.7%)	1257(26.0%)
AATG	AB	AB	134(12.5%)	319(11.6%)	84(10.5%)	211(10.2%)	218(11.7%)	530(11.0%)
Total	-	-	1069(100%)	2761(100%)	801(100%)	2068(100%)	1870(100%)	4829(100%)

Table S2 Agglutination tested blood group vs. genotype-derived blood group

Genotype-derived	Agglutination test					*r*	*P*
	A	AB	B	O	Total		
A	62	0	0	1	63		
AB	0	33	0	0	33		
B	0	0	66	0	66	0.998	<0.001
O	0	0	0	59	59		
Total	62	33	66	60	221		

Table S3 Baseline characteristics of ovarian cancers and controls included for multivariate logistic regression analysis

Variables	Case(N=452)	Control(N=2761)	*P*
Age †	53.33±11.25	53.48±7.72	0.782
BMI (kg/ m^2)			0.927
<18.5	11(2.4)	55(1.99)	
18.5-23.9	194(42.9)	1188(43.03)	
24.0-27.9	171(37.8)	1060(38.39)	
≥28.0	70(15.5)	444(16.08)	
Missing data	6(1.3)	14(0.51)	
First-degree family cancer history			0.941
Yes	337(74.6)	627(22.71)	
No	106(23.5)	2079(75.30)	
Missing data	9(2.0)	55(1.99)	
Age of menarche			0.761
≤15	285(63.1)	1783(64.58)	
＞15	160(35.4)	969(35.10)	
Missing data	7(1.5)-	9(0.33)	
No. of children			**0.034**
Nullipara	19(4.2)	71(2.6)	
1+	404(89.4)	2625(95.1)	
Missing data	29(6.4)	65(2.4)	
Menopause			0.803
Yes	140(31)	908(32.89)	
No	288(63.7)	1817(65.81)	
Missing data	24(5.3)	36(1.30)	
Tubal ligation			**<0.001**
NO	422(93.4)	2111(76.46)	
Yes	30(6.6)	413(14.96)	
Missing data	-	237(8.58)	

†: mean ± s.d.

Bold value indicates p <0.05

Table S4 Baseline and clinical features of 70 EOC patients for IHC staining of antigen A and B

	O	A	B	AB	Total	*P*
N	19(27%)	17(24%)	20(29%)	14(20%)	70	
Age	51.42±7.46	55.06±8.55	48.85±6.94	57.71±11.32	52.83±8.95	0.018
First-degree family cancer history						0.943
Yes	7(36.8%)	5(29.4%)	7(35%)	4(28.6%)	23(32.9%)	
No	12(63.2%)	12(70.6%)	13(65%)	10(71.4%)	47(67.1%)	
Age of menarche						0.464
≤15	15(78.9%)	10(58.8%)	11(57.9%)	10(71.4%)	46(66.7%)	
＞15	4(21.1%)	7(41.2%)	8(42.1%)	4(28.6%)	23(33.3%)	
Menopause						0.459
Yes	7(38.9%)	3(18.8%)	8(42.1%)	4(28.6%)	22(32.8%)	
No	11(61.1%)	13(81.3%)	11(57.9%)	10(71.4%)	45(67.2%)	
Histologic subtypes						0.733
Serous	12(63.2%)	12(70.6%)	10(50%)	8(57.1%)	42(60%)	
Mucinous	1(5.3%)	1(5.9%)	1(5%)	0(0%)	3(4.3%)	
Endometrioid	4(21.1%)	3(17.6%)	8(40%)	5(35.7%)	20(28.6%)	
Clear Cell	0(0%)	1(5.9%)	0(0%)	0(0%)	1(1.4%)	
Others	2(10.5%)	0(0%)	1(5%)	1(7.1%)	4(5.7%)	
Ascites						0.690
None	8(44.4%)	4(28.6%)	3(15%)	2(15.4%)	17(26.2%)	
<500 ml	3(16.7%)	5(35.7%)	7(35%)	5(38.5%)	20(30.8%)	
500-3000 ml	5(27.8%)	4(28.6%)	7(35%)	5(38.5%)	21(32.3%)	
>3000 ml	2(11.1%)	1(7.1%)	3(15%)	1(7.7%)	7(10.8%)	
FIGO tumor stage						0.486
I	5(26.3%)	1(5.9%)	2(10%)	5(35.7%)	13(18.6%)	
II	5(26.3%)	4(23.5%)	4(20%)	2(14.3%)	15(21.4%)	
III	7(36.8%)	10(58.8%)	12(60%)	7(50%)	36(51.4%)	
IV	2(10.5%)	2(11.8%)	2(10%)	0(0%)	6(8.6%)	
Grade						0.869
I	2(11.8%)	1(7.1%)	0(0%)	2(14.3%)	5(8.3%)	
II	7(41.2%)	6(42.9%)	6(40%)	5(35.7%)	24(40%)	
III	8(47.1%)	7(50%)	9(60%)	7(50%)	31(51.7%)	
Adjuvant chemotherapy						0.523
Yes	9(47.4%)	7(41.2%)	5(25%)	5(35.7%)	26(37.1%)	
No	10(52.6%)	10(58.8%)	15(75%)	9(64.3%)	44(62.9%)	

Table S5 Results of IHC staining of antigen A and B in ovarian tumor and adjacent normal tissues (n = 70)

Blood group	Anti-A		Anti-B		No. of patients	Expression patterns
	Para-	Tumor	Para-	Tumor		
O(n=19)						
	-	-	-	-	15	Maintain expression
	-	-	-	+++	4	Incompatible expression B antigen
A(n=17)						
	+++	+++	-	-	3	Maintain expression
	++	-	-	-	1	Loss of expression A antigen
	+++	-	-	-	4	Loss of expression A antigen
	-	-	-	-	2	Loss of expression A antigen
	+++	++	-	-	1	Reduced expression A antigen
	+++	+++	-	+++	1	Incompatible expression B antigen
	+++	+++	-	++	2	Incompatible expression B antigen
	+++	-	-	+++	2	Loss of expression A antigen and incompatible expression B antigen
	-	-	-	+++	1	Loss of expression A antigen and incompatible expression B antigen
B(n=20)						
	-	-	+++	+++	10	Maintain expression
	-	-	++	++	1	Maintain expression
	-	-	-	+++	1	Maintain expression
	-	-	++	+++	2	Maintain expression
	-	-	++	-	1	Loss of expression B antigen
	-	-	+++	++	4	Reduced expression B antigen
	-	-	++	+	1	Reduced expression B antigen
AB(n=14)						
	+++	+++	++	++	3	Maintain expression
	+++	+++	++	+++	1	Maintain expression
	+++	-	++	++	1	Loss of expression A antigen
	+++	-	++	+++	1	Loss of expression A antigen
	+	-	++	+++	1	Loss of expression A antigen
	+++	++	+++	+++	1	Reduced expression A antigen
	+++	++	++	+++	1	Reduced expression A antigen
	+++	++	++	-	1	Loss of expression B antigen and reduced expression A antigen
	++	-	+++	-	1	Loss of expression A and B antigen
	+++	-	++	-	1	Loss of expression A and B antigen
	-	-	++	-	1	Loss of expression A and B antigen
	+	-	+++	++	1	Loss of expression A antigen and reduced expression B antigen

-:negative ; +:weakly positive; ++: moderately positive;+++: strongly positive .

Table S6 Number of patients reduced or lost the expression of appropriate antigens according to blood group type

Blood group	Appropriate antigen expression	Aberrant antigen expression	*P*
O(n=19)	15(78.9%)	4(21.1%)	<0.001
A(n=17)	3(17.6%)	14(82.4%)	
B(n=20)	14(70.0%)	6(30.0%)	
AB(n=14)	4(28.6%)	10(71.4%)	

Table S7 Number of patients reduced or lost the expression of appropriate antigens according to antigen (antigen A: patients with blood group A or AB; antigen B: patients with blood group B or AB)

Antigen	Appropriate expression	Loss of expression	*P*
A (n=31)	10(32.3%)	21(67.7%)	0.004
B(n=34)	23(67.6%)	11(32.4%)	

Table S8 Baseline and clinical characteristics of 14 patients for gene expression profile

Blood group	O	A	B	AB	Total	*P*
N	2(14.3%)	7(50.0%)	4(28.6%)	1(7.1%)	14(100%)	-
Age	55±9.9	56±4.5	58±5.5	76	58±7.3	0.345
Age of menarche						0.610
≤15	2(100%)	4(57.1%)	2(66.7%)	1(100%)	9(69.2%)	
＞15	-	3(42.9%)	1(33.3%)	-	4(30.8%)	
Menopause						0.405
Yes	1(50%)	6(85.7%)	4(100%)	1(100%)	12(85.7%)	
No	1(50%)	1(14.3%)	-	-	2(14.3%)	
FIGO tumor stage						0.397
I	-	1(14.3%)	-	-	1(7.1%)	
II	1(50%)	3(42.9%)	1(25%)	-	5(35.7%)	
III	1(50%)	2(28.6%)	3(75%)	-	6(42.9%)	
IV	-	1(14.3%)	-	1(100%)	2(14.3%)	

Table S10 Baseline and clinical characteristics of 116 patients for RNA-seq and methylation profile

Blood group	O	A	B	AB	Total	*P*
N	28(24.1%)	41(35.3%)	29(25%)	18(15.5%)	116	-
Age	55.25±12.47	53.88±8.99	52.55±6.98	54.72±5.23	54.01±9.03	0.709
Age of menarche						0.907
≤15	18(64.3%)	24(58.5%)	19(65.5%)	12(66.7%)	73(62.9%)	
＞15	10(35.7%)	17(41.5%)	10(34.5%)	6(33.3%)	43(37.1%)	
Menopause						0.339
Yes	12(44.4%)	14(35%)	14(48.3%)	4(23.5%)	44(38.9%)	
No	15(55.6%)	26(65%)	15(51.7%)	13(76.5%)	69(61.1%)	
FIGO tumor stage						0.304
I	4(14.8%)	6(15.8%)	0(0%)	3(16.7%)	13(11.6%)	
II	5(18.5%)	9(23.7%)	7(24.1%)	5(27.8%)	26(23.2%)	
III	16(59.3%)	16(42.1%)	20(69%)	9(50%)	61(54.5%)	
IV	2(7.4%)	7(18.4%)	2(6.9%)	1(5.6%)	12(10.7%)	
Histologic subtypes						0.597
Serous	17(60.7%)	23(56.1%)	16(55.2%)	11(61.1%)	67(57.8%)	
Mucinous	1(3.6%)	2(4.9%)	1(3.4%)	-	4(3.4%)	
Endometrioid	6(21.4%)	10(24.4%)	11(37.9%)	5(27.8%)	32(27.6%)	
Clear Cell	2(7.1%)	-	-	1(5.6%)	3(2.6%)	
Others	2(7.1%)	6(14.6%)	1(3.4%)	1(5.6%)	10(8.6%)	
